# The constraints of antiretroviral uptake in rural areas: the case of Thamaga and surrounding villages, Botswana

**DOI:** 10.1080/17290376.2014.972057

**Published:** 2014-11-03

**Authors:** Matlhogonolo Bene, Michael B.K. Darkoh

**Affiliations:** ^a^ BA Environmental Science, MA Geography, is affiliated to the Department of Environmental Science, University of Botswana, Private Bag 708, Gaborone, Botswana; ^b^ Professor of Environmental Science, is affiliated to the Department of Environmental Science, University of Botswana, Private Bag 708, Gaborone, Botswana

**Keywords:** rural areas, HIV/AIDS, ARV uptake, Masa programme, Zones rurales, VIH/SIDA, prise des ARV, programme Masa

## Abstract

This article examines the constraints of antiretroviral (ARV) uptake in the villages of Thamaga, Kumakwane, Mankgodi and Gakgatla which are in the Kweneng District of Botswana. The social interactionist approach and theories of health behaviour provided the theoretical basis of the study. Data were obtained by using interviewer-administered questionnaires which were applied to a sample of 145 respondents and 61 people living with HIV/AIDS in the four villages. The results of the study showed that people aged 30–39 years represented the highest proportion of the persons on ARV treatment in the villages. Some of the people living with HIV believed that ARV therapy could better their lives during the initial stages of introduction, but with time, they lost hope and gave up the treatment. Culturally, parents and children in the villages do not discuss sexual matters at home and it was found in the study that there was little communication between parents and children on AIDS and ARV issues. Some churches in the area discouraged the use of ARV. There were also traditional doctors who made their patients mix traditional herbs treatment with ARV treatment. Distance, travel costs, cultural beliefs, stigma and discrimination among others were found to be important socio-economic factors inhibiting ARV uptake. Even though there were constraints on ARV uptake in the villages, efforts were being made by Government and non-governmental organizations to overcome them. The Ministry of Health provided information and education to the public using its strategy known as Information, Education and Communication. Nurses, doctors and chiefs taught people at kgotlas (traditional courts) in the villages about the dangers of the epidemic. Free HIV testing, ARVs and condoms were provided to the villagers. The outlook for ARV uptake looks generally promising for the future. However, if HIV/AIDS is to be contained, sexual behaviour of people in the villages needs to change.

## Résumé

Cet article examine les contraintes liées à la prise des ARV dans les villages de Thamaga, Kumakwane, Mankgodi et Gakgatla dans le district de Kweneng au Botswana. L'approche interactionniste et les théories sur le comportement face à la santé ont constituées la base théorique de cette étude. Les données ont été récoltées au moyen de questionnaires administrés par l'enquêteur appliqués à un échantillon de 145 répondants et 61 personnes vivant avec le VIH/SIDA dans les quatre villages. Les résultats de l’étude montrent que les personnes âgées de 30 à 39 ans représentent le plus grand nombre de personnes sous traitement antirétroviral (ARV) dans les villages. Certaines des personnes vivant avec le VIH croyaient que la thérapie ARV pourrait améliorer leur vie pendant les phases initiales de son utilisation mais avec le temps, ils ont perdu espoir et ont abandonné le traitement. Sur le plan culturel, les parents ne discutent pas des questions sexuelles avec les enfants à la maison et l’étude à révélé qu'il y avait peu de communication entre les parents et les enfants sur les questions du SIDA et les ARV. Certaines églises de la région décourageaient l'utilisation des ARV. D'autre part, des médecins traditionnels ont fait prendre aux patients un traitement à bases d'herbes traditionnelles en même temps que le traitement ARV. La distance, les frais de voyage, les croyances culturelles, la stigmatisation et la discrimination se sont révélés être des facteurs socio-économiques importants entravant la prise des ARV. Malgré ces contraintes liées à la prise des ARV dans les villages, le gouvernement et les ONG déploient de nombreux efforts pour les surmonter. Le ministère de la Santé informe et éduque à travers sa stratégie Information, Éducation et Communication (IEC). Dans les *kgotlas* (cour traditionnelle) des villages, les infirmiers, les médecins et les chefs informent la population des dangers de l’épidémie. Le dépistage du VIH, les ARV et les préservatifs sont fournis gratuitement aux personnes dans les villages. De façon générale, les perspectives de la prise des ARV semblent prometteuses pour l'avenir. Cependant, si le VIH/SIDA doit être maitrisé, le comportement sexuel des personnes dans les villages doit changer.

## Introduction

Since it was first discovered over 30 years ago, HIV has become one of the most devastating epidemics, taking the lives of 30 million people around the world (One International 2011). It is estimated that globally, in 2010 alone, HIV/AIDS killed 1.8 million people, of whom about 67% (1.2 million) were living in Sub Saharan Africa (ibid). The HIV/AIDS epidemic has already reversed many of the development gains made in Central, Eastern and Southern Africa over the past three decades (UNDP 1997). Though life-saving antiretroviral (ARV) treatment is available, access is not yet widespread in many countries of Sub Saharan Africa. By 2010, more than 6.6 million people were on life-saving ARV treatment of whom more than 5 million were living in Sub Saharan Africa (One International 2011). Even more worrying, new HIV infections have continued to outpace those added onto ARV treatment (One International 2011).

Botswana has one of the highest HIV infection rates in the world, with a general population prevalence of 19.8% in women and 13.9% in men (Ministry of Health [MOH] 2004). More alarming, infection rates are much higher in vulnerable groups such as pregnant women, at 32.4% (Government of Botswana 2006) and men on separation in marriage, at almost 70% (Mosarwe 2014). The government of Botswana, confronted by this major national crisis, decided to explore the feasibility of making ARV therapy available nationally. It forged a public–private partnership in July 2000 with the African Comprehensive HIV/AIDS Partnerships (ACHAP), an non-governmental organization (NGO) formed by the Bill & Melinda Gates Foundation and The Merck Company Foundation/Merck & Co., Inc to combat the scourge (MOH 2005). As part of this partnership, both Bill and Melinda Gates and the Merck Company Foundation contributed US$50 million each, while Merck & Co., Inc., pledged to donate two types of ARV medicines free of charge for the duration of the partnership (MOH 2005). Out of this partnership was born the ‘Masa programme', which is the brand name for the national ARV programme. As the first African country to implement a full-scale national ARV programme, Botswana has become a beacon of hope for the rest of the continent suffering from AIDS. The name of the national ARV Programme, ‘Masa', which means ‘new Dawn' in Setswana, ‘signifies the hope that ARVs offer to people living with HIV and AIDS, which includes the hope to live longer, healthier lives by providing them with time to nurture their families and build a future for the nation' (Government of Botswana 2006:3).

The overall goal of the Masa programme was to enhance prevention efforts and reduce the impact of HIV/AIDS on the people of Botswana through the introduction and effective utilization of ARV therapy. The objectives of the programme were to (Government of Botswana 2006):

- Mobilize communities to support treatment and care of HIV infected individuals.
- Build capacity, both human resource and infrastructure, necessary for the successful implementation of the programme.
- Provide highly active antiretroviral therapy (HAART) to eligible individuals.
- Advise on and implement systems and policies to support the ARV therapy programme.
- Guide/coordinate all efforts in the country geared towards implementation of the ARV programme.
- Monitor and evaluate the programme to ensure continuous improvement of quality and effectiveness.

**Fig. 1.**
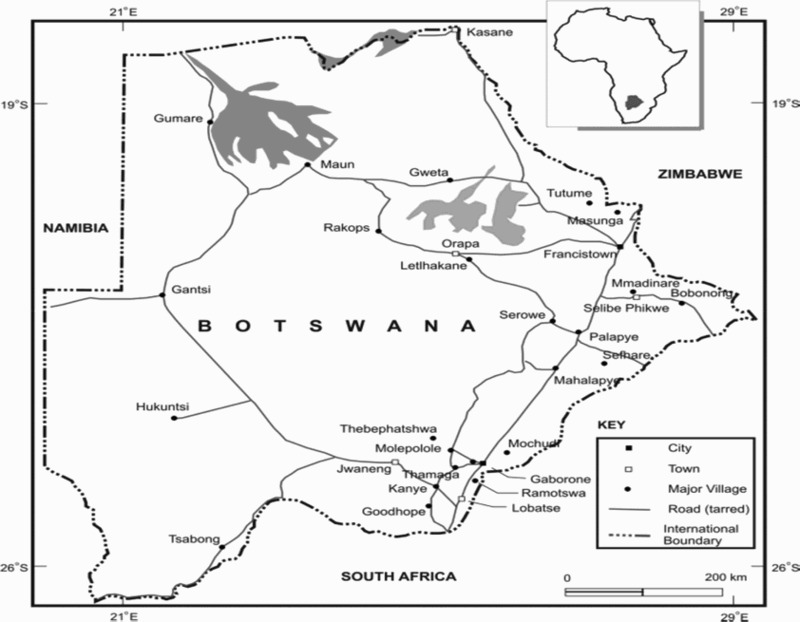
ARV sites in Botswana.

Provision of free treatment was built around an ‘ART site’ model, consisting of a hospital supported by satellite screening clinics. From January 2002 onwards, ARV therapy (ART) sites had been rolled out in a phased manner and 32 ART sites and 178 satellite clinics across the country were dispensing ARVs by the end of June 2010. Botswana Public Officers Medical Aid Scheme, Pula, Botswana Medical Aid Society and Private Medical Care and Workplace programmes have been monitoring and reporting on ARV use quarterly for the private sector, while Associated Fund Administrators has been responsible for outsourced clients (Ministry of Health [MOH] 2008). Fig. 1 shows the ARV sites in Botswana.

From an initial rollout to the major sites at Gaborone, Serowe and Maun, the National ARV Programme has expanded and by January 2008, all 29 Public hospitals and over 60 local clinics in the country had been equipped with ARV facilities. In 2008, out of an estimated 130,000 people eligible for treatment, over 100,000 people had been put on life-saving ART across the nation (Ministry of Health [MOH] 2008). By the end of June 2010, a total of 123,474 infected people were on treatment in the public sector in the country, of whom 61.5% were females, 31.9% males and 6.6% children (MOH 2008). A further 15,412 were treated by the private sector under the Government's Out-sourcing Programme and 12,809 other people on ARVs were being treated in the private sector of the country by the Medical Aid Schemes and Work-place Programmes. This gives a total of 151,695 people currently receiving HAART in Botswana. This accounts for 92.6% of the projected total of 163,872 adults and children in need of ART at the end of June 2010 (MOH 2010). Since the inception of ARV in the country, a cumulative total of 16,016 people on ARVs had died while on HAART (MOH 2008).

Even though steps are being taken by the government and international agencies to curb the escalating HIV/AIDS epidemic in the country, through free provision of ARVs and information on assessing and reducing the risk of infection, the epidemic is still spreading in Thamaga and its surrounding villages as it is in other parts of the country and people are dying. The main motivation for this article is to find out what are the major socio-cultural constraints to ARV uptake which is leading to the continuing demise of people. This article therefore examines the major factors inhibiting ARV uptake in the villages. The general hypothesis is that sociocultural factors are constraining ARV uptake in the villages. The sociocultural factors examined include public attitude towards HIV patients on ARV, religion, cultural beliefs, stigma and discrimination, child–parent dialogue and distance travelled to acquire ARVs.

## Theoretical basis and literature review

### Theoretical basis

Two theoretical approaches underpinned this study. These were the theories of health behaviour and the social interactionist/humanist approaches. Theories of health behaviour were used to explore the villagers' behaviour towards ARV treatment. Two main models of health behaviour were used. These were the health belief model and the theory of reasoned action. The health belief model is based on the understanding that a person will take a health-related action if the person feels that a health condition can be avoided, has a positive expectation that by taking a recommended action, he/she will avoid a negative health condition and believes that he/she can successfully take a recommended health action (Gatrell 2002; Vilili 2008). The model attempts to explain and predict health behaviours by focusing on the attitudes and beliefs of individuals, particularly with respect to their perceived susceptibility, seriousness and severity of the disease, benefit of service and barriers to accessing health care (Azjen & Fishbein 1980). This theory guided the design of the questionnaire and the analysis of the findings.

The theory of reasoned action says that a person's behaviour is determined by his/her attitude, the outcome of that behaviour and by the opinions of the person's social environment (Azjen & Fishbein 1980). The theory defines an attitude as a person's innate belief about whether the outcome of his/her action will be positive or negative while subjective norms are beliefs about what others would think about the behaviour. The theory of reasoned action was used to assess whether ARV was regarded as a social norm by the villagers or not and to determine their attitude towards ARV therapy. It was also used in the study to understand the underlying structures in the village community which hasten or hinder individuals seeking medical help in various health facilities when they fall sick.

The social interactionist/humanist approach places emphasis on the meaning of an illness or disease to an individual and understanding and meanings that make it rational to act in a particular way. The approach believes that if people do not see the severity in a particular disease, they may not be bothered to seek treatment (Gatrell 2002; Vilili 2008). Social interactionist approaches study individuals, small numbers of people, small communities such as villages or neighbourhoods rather than studying large areas. This is because the experience of place is more important than the accurate recordings of large numbers of locations (Gatrell 2002; Vilili 2008). The methods used are essentially qualitative rather than quantitative and the ultimate goal is empathic understanding and explanation rooted in the social, rather than the natural world (Gatrell 2002). The social interactionist approach was used in this research to understand what ARV therapy means to the infected people and the villagers in general.

## Literature review

Few studies are available on ARV uptake in Africa. This section of the paper reviews the relevant literature on ARV uptake with special reference to Southern Africa, including Botswana.

A study on equity and access to ARV drugs in Malawi was conducted using a qualitative methodology (Ntata 2007). Interviews for the study covered all the three regions of Malawi. The study found that information benefited only people who were physically and socially nearer to ARV administration points. Physically, those in remote areas were disadvantaged as there were no newspapers and radios. Socially, information was accessible to those who were educated. Health services in Malawi were not evenly distributed and mostly concentrated in urban areas. For the poor and those living far away from Voluntary Counseling and Testing Centers and ARV clinics, availability and affordability of transport were key issues with regard to accessing the services. The study found that people were failing to travel to the district or central hospitals for ART initiation which required at least two visits.

Another major study on Malawi (Vilili 2008) dealt with the geographical analysis of ARV uptake. The study concentrated on the uptake among the youth in Lilongwe. Based on a sample of 1206 infected youth aged 15–24 who were on ART in Lilongwe Rural and Urban, the study analysed the proportion of the youth on ARV treatment, the openness about HIV status and ARV treatment, distance travelled to acquire ARV treatment and the perceptions of the youth. The study found that only 8.8% of the sample was on ARV treatment and 43% disclosed their HIV status to their family members. The youth had problems in discussing their status with friends, church leaders and teachers. The study noted that while those in Lilongwe Urban travelled shorter distances of 2–5 km, those infected in Lilongwe Rural had to travel more than 5–6 kms for ARV treatment. It also found that in several cases, the youth were prevented by social stigma from accessing ARV treatment. This last point corroborates with the finding of the study outlined above (Ntata 2007) which also noted that revealing one's status in Malawi could be risky and costly especially for women since men could force their wives to leave the house if they were found to be HIV-positive.

A study on the challenges of ART treatment in Tanzania noted that perceptions about ARVs varied among users (Dennis, Wasai & Ellen 2007). Some of the respondents in the study indicated that ARVs had significant side effects, while others said that ARVs were helpful in ameliorating the disease in the body. Several infected men believed that if they stopped the medication, their bodies would go weak, while most of the women indicated that, taking ARVs, kept the virus at bay and lengthened the user's life (Libeon 2007).

In a study cited by Vilili (2008) on the experiences of ARV uptake in the provinces of Kwa-Zulu Natal and Limpopo, South Africa, the main factor found was stigma which prevailed in the HIV/AIDS care, treatment and even prevention. People living with AIDS at both household and community levels were reported to have difficulties in disclosing their status in fear of discrimination. Disclosure of status was often linked to the advanced stages of AIDS, that is, most people disclosed their status just before death.

Before the introduction of ARV therapy in 2002 in the national healthcare system, the Information, Education and Communication (IEC) environment in Botswana was largely based upon community outreach and mass media, promoting HIV/AIDS awareness and prevention messaging. Over the years, several surveys had been conducted to measure the efficiency of these campaigns. Most of the results had showed that there was a high level of awareness of HIV/AIDS within the communities. However, the Ministry of Health (MOH) found that the steady increase in HIV prevalence in Botswana during that period indicated that people were not translating knowledge and awareness about HIV/AIDS into changing behaviours (MOH 2005).

An article on the challenges of ARV treatment in Botswana indicated that out of 950 people on ARV treatment in the Kgalagadi district, about half resided in remote villages outside the district headquarters, Tsabong, where ARVs were dispensed (Seleke 2008). After realizing that many people in these areas were unemployed with little or no income and no transportation, district officials pushed for the rollout of treatment to satellite clinics (ibid). The factors that affected the ARV uptake which were identified included distance and transport costs.

The site location of clinics is an important role in access of ARVs (Jones 2012). Patients who stayed far and in remote areas sometimes skipped the medication as well as monthly appointments because they could not afford transport costs. The same trend was found in a study in Botswana where about 13% of patients indicated that travel or migration were the main reasons for missed medical appointments and pill doses (Taylor, Garduno, Reyes, Rojas, Donastorg, Brudney, *et al*. 2011).

In another study on ARV uptake in Botswana (Nam, Fielding, Avalos, Dickinson, Gaolathe & Geisler 2008), it was found that the principal barriers to ARV adherence included financial constraints (44%,) stigma (15%,) travel/migration (10%) and side effects (9%). If costs were removed as a barrier, adherence was predicted to increase from 54% to 74% (Nam *et al*. 2008). The study was done at the national level and did not indicate what was happening at the rural level. The other drawback was that the study focused only on the youth.

From the foregoing, much of the available literature on ARV uptake is topical and pitched at the macro scale of spatial analysis, that is, at the national level. Studies which analyse ARV uptake at local and community levels are few. To date, in Botswana, there are no outstanding studies on ARV uptake at the rural or village level. It is hoped that the findings of this study will contribute to fill the current gap in the literature.

## Methods

The study is descriptive and used a mixed-method approach to collect and analyse data in order to understand ARV uptake in the villages. Both qualitative and quantitative methods were used in the study.

### Qualitative and quantitative approaches

The use of the descriptive qualitative approach is in line with the humanist/social interactionist approach which emphasizes getting the meaning of the illness or disease rather than the magnitude of the situation (Gatrell 2002; Vilili 2008). In conducting the study, qualitative methods were largely used in order to uncover and interpret the meaning of the illness which makes it ‘rational’ for patients to act in a particular way (Gatrell & Elliot 2009:84). According to Creswell (1994), people are interested in meaning, how people make sense of their lives. The study used in-depth interview guide to get the qualitative data from the clients.

However, in order to get the magnitude of the issues being discussed, quantitative methods in the form of descriptive statistics such as Chi square and ANOVA were used to complement the findings. The mixing of both qualitative and quantitative methods, as Gatrell and Eliot (2009:84) note, has proved singularly useful, with insights from in-depth interviews adding colour and explanatory power to the quantitative to shed light on the research questions.

### Sampling

**Fig. 2.**
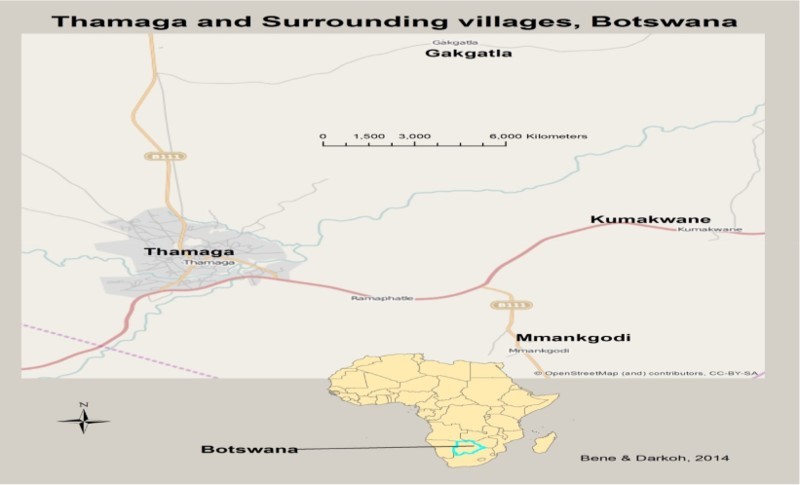
Study area.

Preceding the determination of sample size, a pilot reconnaissance survey was carried out with the assistance of health workers in Thamaga hospital and clinics in the area, to select the villages to be included in the sample. Three villages were selected in addition to Thamaga for the field study. These villages were Mankgodi, Kumakwane and Gakgatla (Fig. 2).

To calculate the sample size, the following formula was used: **n = N/1 + N (e)^2^,** whereby **n** equals sample size, **N** equals number of households and **e** equals marginal error. Altogether, 145 respondents (95% confidence level, 5% error) were selected and interviewed. Stratified random sampling was used to select respondents from the four villages.

#### Purposive sampling

In order to gain further insights into the disease and patients' experience and constraints of ARV uptake, purposive sampling was used to select 61 patients out of a total of 3572 people in the four villages living with HIV. These 61 patients were the only ones who agreed to partake in the exercise. The researchers administered in-depth interviews to these patients. During data collection, the researchers used a tape recorder and note-taking so that all information given by the clients was captured. Both structured and semi-structured questions were used in the individual interview guide.

#### Ethical consideration

Before the field data collection, ethical permit was sought and obtained from the Institutional Review Board, an organization in the MOH, Botswana, which deals with ethical matters. Informed consent was sought from the clients. Clients were made clear that participation was entirely voluntary and that they were free to withdraw from the study at any point in time.

#### Data collection

Quantitative data collection took 6 months, from July to December 2010. Interviewer-administered questionnaires were the main tools used in collecting primary data. These data were obtained from three main sources which were the general public or residents of Thamaga and surrounding villages, people who were HIV-positive and on ARVs and key informants. The interviews were conducted face to face. Interviews with the general public were carried out in order to gain an understanding of local residents' attitudes towards people living with HIV/AIDS and their overall views on ARV. Secondary data were collected from published and unpublished sources. ARV registers and records acted as the principal source of secondary data. They were used to ascertain the people who were HIV-positive and on ARVs. These records were collected from the MOH in Gaborone and the Thamaga Hospital and clinics in the surrounding villages. Table 1 is a summary of the objectives, research questions and methods used in the data collection.

**Table 1.** Summary of objectives, research questions and methods of data collection and analysis.

Objectives	Research questions	Methods of data collection	Tools of data analysis
1. To examine the extent of ARV uptake in these villages	How do age and gender influence ARV uptake among the infected?	Tracking people on ARV with aid of health workers	Use of SPSS, Bar graphs, Microsoft Excel. Listing responses from survey data
		• 95% significance level established	
2. To find out the socio-economic factors which inhibit or constrain ARV uptake in these villages	How do public attitudes, culture, religion, lack of dialogue between parents and children and residence or distance affect the uptake of ARV in Thamaga and surrounding villages?	Use of questionnaires and interviews (with the residents or the public, key informants and people on ARV)	Description: listing responses from survey data. Use of Frequencies, SPSS and Bar graphs

Data were gathered, using open-ended and closed questions. Also, before conducting the main interviews, a pilot study was also carried out in order to test the accuracy of the questionnaires. The two primary investigators did the self-administration of questionnaires with no external assistants.

#### Key informants

People on ARV were central to this study and to track them key informants were indispensable. Key informants (16) were drawn mainly from health workers and doctors in the Thamaga Hospital and village clinics in the surrounding areas. Other key informants (4) of the study included officials from the MOH in Gaborone and NGOs such as United Nations Agency on AIDS, National AIDS Coordination Agency and ACHAP. These key informants were chosen because the researchers believed them to be knowledgeable on the issues being investigated. The health workers (12) were of particular assistance in tracking down the people when they came for their ARVs in the hospital and clinics.

### Data management and data analysis

The data were coded, keyed and descriptively analysed through Statistical Package of Social Sciences (SPSS) and Microsoft Excel. Descriptive methods of statistical analysis were used, as indicated already. The findings were presented in the form of cross-tabulations, tables and pie charts.

## Findings

### Extent of ARV uptake

Table 2 shows the age and sex distribution of people on ARV in Thamaga and surrounding villages. From the table, we can observe that females of all ages were dominant.

**Table 2.** Age and sex distribution of patients on ARVs in Thamaga and surrounding villages, 2009.

Age range (years)	Females at Thamaga hospital	Females in surrounding clinics	Males in Thamaga hospital	Males in surrounding clinics	Total	Percentage
0–4	17	6	15	2	40	1.12
5–9	17	0	19	2	38	1.06
10–14	15	5	15	1	36	1.01
15–19	4	11	7	3	25	0.70
20–24	21	101	2	15	139	3.89
25–29	101	273	23	77	474	13.27
30–34	164	325	84	179	752	21.05
35–39	146	199	127	191	663	18.56
40–44	116	126	96	132	470	13.16
45–49	87	80	86	107	360	10.08
50–54	61	45	89	55	250	7
55–59	30	27	55	36	148	4.14
60+	45	33	45	46	169	4.73
Age unknown	2	2	2	2	8	0.22
Total	826	1233	665	848	3572	100

Source: MOH (2009).

People in the age group of 25 and above showed a high percentage of people on ARVs. The age groups with the highest percentages were 30–34 and 35–39 with respectively 21.05% and 18.56%. Table 3 shows the recorded number of people collecting their ARV from clinics in the study villages in 2010. Mmankgodi clinic recorded the lowest number of patients, while Nkoyaphiri had the highest number of patients. This could be due to the fact that Nkoyaphiri is nearer to Mogoditshane, a densely populated surburb of Gaborone, the national capital. Most patients who live in Gaborone and its suburbs who might be afraid to go for therapy in the city where people know them might go to clinics far away such as Nkoyaphiri.

**Table 3.** Number of people collecting ARVs from clinics in the study villages in 2010.

ARV site	Total number of patients
Thamaga primary hospital	1646
Nkoyaphiri clinic	1726
Gabane clinic	392
Thamaga clinic	114
Mmankgodi clinic	70
Kopong clinic	200
Mmopane clinic	120
Total	4268

Source: MOH (2010).

### Constraints to ARV uptake

As noted, the theory of reasoned action says that a person's behaviour is determined by, among other things, the opinions of the person's social environment (Azjen & Fishbein 1980). Different people would always react differently. One of the social factors assumed to inhibit ARV uptake was public attitude and to verify this, respondents from the general public were asked how they would treat people with HIV who were on ARVs (Table 4).

**Table 4.** How would you treat someone who is taking ARVs?

	Frequency	Percent	Cumulative per cent
With love and respect	68	46.9	46.9
Do not want anything to do with them	11	7.6	7.6
Treat like any other person	66	45.5	45.5
Total	145	100.0	100.0

The results showed that 46.9% respondents would not shun them and would treat them with love and respect. About 45.5% indicated that they would not discriminate against people on ARVs, while 7.6% indicated that they would not want to have anything to do with them as they were sick. The evidence pointed to villagers gradually starting to realize that HIV/AIDS is like any other disease. ‘These people are like any other people, who are we to judge others?' said one respondent. Another respondent from survey said ‘HIV positive persons are sinful people. They go around sleeping with everyone and the next thing they want is mercy from us. No! They should live in their own world'.

Religion is another socio-cultural factor that plays an important role in the lives of villagers whose influence on ARV uptake was investigated. Table 5 shows religion and its effects on ARV uptake. There were a large number of respondents (55.9%) who attended churches that encouraged the use of ARVs. About 12% went to churches that discouraged the use of ARVs, while 11% went to churches that did not discuss about the use of ARVs. Out of the total sample of 145, 0.7% said that their religion (Catholic) allowed ARVs but emphasized abstinence; another 0.7% said that their religion encouraged the use of ARVs and traditional medicines. About 13% did not belong to any religious denomination and therefore had no views to express on the influence of religion on ARV uptake. The results were further evaluated to see if there is a statistical difference in how the respondents from religions that encouraged and discouraged the use of ARVs would treat people who were on ARVs. Chi-square (*χ* ^2^) was used since the variables were categorical. In testing this relationship, a positive association was found (*χ* ^2^ = 118.045, df = 2, Phi = 0.902 *P* > 0.01). Most of the respondents said that they would treat HIV people with love and respect. The majority of those respondents (57%) who said that they would treat HIV patients with love and respect went to churches that encouraged the use of ARVs, while only 2% went to churches that do not (Table 6). Respondents who were members of churches that discouraged the use of ARVs were mostly the Zezurus who believed that God is the creator and the healer. With this belief, they do not even take their sick relatives to the clinics when they are ill. This discourages the government's efforts of trying to bring facilities closer to people. Since churches like the Catholics encourage the use of ARVs, but emphasize abstinence, if a member of the church had sex outside marriage and contracted the disease, they would invariably hide it from other church members because of the fear of being judged. Most Batswana still believe that traditional doctors can cure HIV/AIDS. So, they sometimes stop the treatment and take on traditional herbs. This evidence was corroborated by key informants, who said that some villagers preferred the use of traditional medicine while others used traditional medicine while on ARVs which was a health risk.

**Table 5.** Effect of religion on ARV.

	Per cent	Cum. per cent
Encourage ARVs	81	55.9
Do no talk about them	16	11.0
Do not know	9	6.2
Discourage them	18	12.4
Partnership between tradition and modern medicine	1	0.7
Not applicable	19	13.1
Allowed but abstinence emphasized	1	0.7
Total	145	100.0

**Table 6.** Effects of religion on ARV uptake.

			How the residents treat people taking ARV treatment	How the residents treat people taking ARV treatment	Total
			Treat with love, and no discrimination	Would not associate with patients on ARVs	
Religion effects on ARV use	Encourage the use of ARVs	Count	82	0	82
		% within religion effects on ARV use	100.0%	0.0%	100.0%
		% of total	56.6%	0.0%	56.6%
	Do not discuss the use of ARVs	Count	45%	0	45
		% within religion effects on ARV use	100.0%	0.0%	100.0%
		% of total	31.0%	0.0%	31.0%
	Discourage the use of ARVs	Count	3	15	18%
		% within religion effects on ARV use	16.7%	83.3%	100.0%
		% of total	2.1%	10.3%	12.4%
Total		Count	130	15	145
		% within religion effects on ARV use	89.7%	10.3%	100.0%
		% of total	89.7%	10.3%	100.0%

**Fig. 3.**
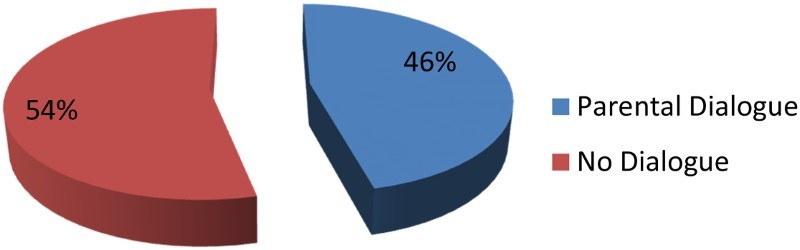
Parental dialogue on HIV/AIDS, ARV issues.

Parent–children dialogue was another social factor identified as inluencing ARV uptake. Respondents were asked whether parents had dialogue with their chidren on ARV uptake. About 46% said that parents talked to their children about ARV issues (Fig. 3). Of the total sample, 54% said that their parents never discussed ARV issues with them. In Setswana culture, communication between parents and children about sexual behavior is not common. This leads to children making mistakes because there is no one to guide them. Parents believed that teachers were doing their job at school when teaching their kids about sexual reproduction. This was what a 15-year-old respondent had to say, ‘my mother would chase me out of the house if I metioned AIDS and ARVs' (respondent from survey data).

During the survey, the practice of breastfeeding was one of the factors identified by key informants from the MOH as influencing and being influenced by ARV uptake. Breastfeeding is one of the significant cultural practices in Botswana and according to the key informants, many mothers feel that a woman who does not breastfeed might not be accepted in the society. Often, if a mother did not breastfeed her child, people tended to suspect that she was HIV-positive and they sometimes stigmatized and called her funny names. Because of this, some HIV patients ended up breastfeeding in order to fit into the social setup.

### The 61 case study patients

**Fig. 4.**
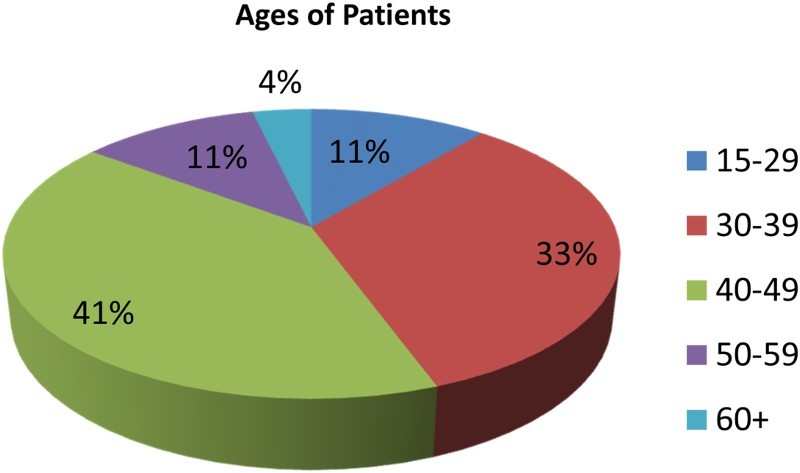
Age of case study HIV patients.

As already stated, patients on ARV were central to this study and 61 of them were purposively selected to get in-depth information on the disease and patients' experience and constraints of ARV uptake. Fig. 4 illustrates the ages of the case study patients. The majority of the patients were aged 40–49 (41%). Those in the ages 30–39 followed at 33%. Patients aged 15–29 and 50–59 each had 11%. Those with the least percentage were the patients aged 60+. Females constituted 59% of the sample and males 41%.

To ascertain whether there were any social inhibitions that would prevent clients from going for ARV treatment, patients were asked whether they were free to go for ARV treatment.

**Fig. 5.**
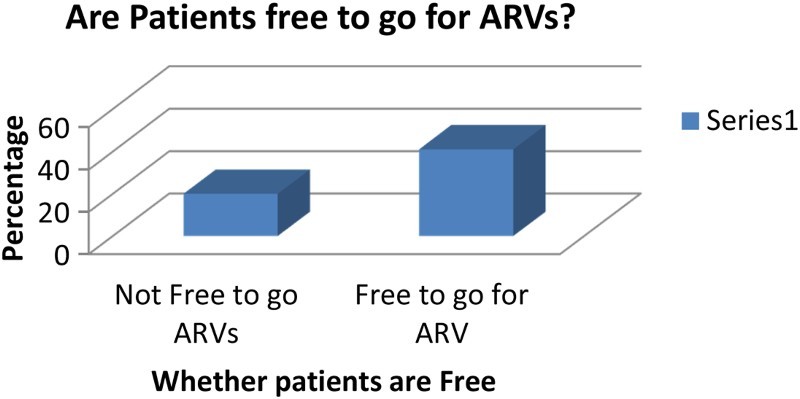
People living with HIV and on ARV.

Fig. 5 shows patients' responses.

Of the 61 respondents, 66.4% said that they were free to go for their medication, while only 33.6% said that they were not free. The reason the latter gave for not being free was that people shunned them because they were infected. People said that they were just waiting to die. This attitude made it difficult for them to go freely for their medication. Most of the people who said they were free to go for their medication explained that it was because they had accepted their status.

Those patients who felt that they were not free to go for AIDS medication said that it was because of the fear of stigmatization and discrimination from society. Another factor mentioned was lack of cooperation from employers who frequently refused to give permission or allow sick leave to the people. Most of the people who were free were those with full-blown AIDS and such people were free because they did not want to die and therefore did not care about what people thought about them. Several of the people on ARV came to Thamaga hospital from places as far as Moshupa, Lobatse, Nkoyaphiri, Gakgatla, Manyana and Kumakwane for collection of their medication and treatment.

During the study, clients on ARV were asked if they had made known that they were on ARV with any one. This was asked to ascertain whether clients were free and open to discuss the subject of HIV and ARV. The study found that 88% of the patients discussed their sero and ARV status with their parents or very close relatives. Another 86% discussed their status with their spouses. About 5% said that they never discussed with any one about their HIV sero status. These findings show that the patients limited the discussion to close family members and spouses and not to other members of society.

The patients were also asked to share the perceptions or reactions of the people to whom they disclosed the fact that they were on ARV. One female patient revealed that when she broached her ARV status to her spouse, he started neglecting her and eventually dumped her for another woman. Another young patient said that he discussed the results wih his siblings and they started calling him all sorts of names and even tried to shun him. The clients who did not discuss their sero and ARV status with anyone pointed out that the main reason was fear or stigma and discrimination.

According to the patients, especially public attitude had continued to be a major problem as they were often shunned by some members of the public who happened to know that they were on ARV. Most often, people stigmatized and discriminated against them. However, according to the patients, the perceptions of parents regarding treatment were generally positive as parents were supportive and encouraged them to take the treatment seriously. The study noted that there were mixed perceptions and attitudes between spouses who were informed about ARV treatment. Some spouses were supportive, but others, uncooperative. One husband chased the wife out of their matrimonial home when he was told about his spouse's ARV treatment. The study found that a primary concern before patients started ARV treatment was fear of the consequeces. Many thought that they would die and several were worried about possible side effects.

Finally, distance was one of the factors identified by patients as inhibiting ARV uptake (Table 7). This factor is important when viewed within the context of patients' affordability of transport costs and/or ability to travel on foot to collect medication.

**Table 7.** Distance travelled by HIV patients to ARV clinics.

Distance	Frequency	Percent
0–0.5 km	32	51.9
1–10 km	11	18.5
More than 10 km	18	29.6
Total	61	100.0

On this aspect, patients were asked about the distance they travelled to obtain their dosages of ARV. About 52% travelled 0–0.5 kms from their places of residence to get their medication, 18.5% travelled 1–10 kms and 29.6%, more than 10 kms. Those staying far from ARV sites included illiterates and poverty-stricken people, who besides being ill-informed, often defaulted. ANOVA was used to test the variances between the distances travelled by the patients (Table 8). Because of poverty and unemployment, some HIV patients walked long distances to collect their ARVs. While some patients walked distances of less than 5 kms for ARVs, some walked more than 10 kms for ARVs (F4.224, df (1, 59) *P* > 0.05). For people who are sick, distance travelled to collect ARVs becomes a critical variable.

**Table 8.** ANOVA on distance travelled by patients.

			Sum of squares	df	Mean square	*F*	Sig.
Between groups	Combined	Unweighted	3.126	1	3.126	4.224	0.044
			3.126	1	3.126	4.224	0.044
			3.126	1	3.126	4.224	0.044
Within groups			43.661	59	0.740		
Total			46.787	60			

About 48% of the patients indicated that travel posed a constraint. They associated long travel with high transport costs and said that they sometimes failed to go to collect their medication because they could not afford the transport costs. The study found that about 15% of the pateints walked on foot from their villages to the health facilities since they could not afford the cost of travel by bus or taxi. Travel therefore posed a big challenge in accessing ART in the rural areas, especially when patients had no money and were sick or weak to easily move about.

To sum up, in view of the foregoing constraints, many of the patients did not feel really free to go for ARVs and were finding life difficult to cope. It would appear that, of all the constraints, public attitude towards HIV patients on ARV, fear, stigmatization and discrimination, parental dialogue, religion. distance and transport affordability were the most important factors constraining ARV uptake.

## Discussion, summary and conclusion

The study has shown that females on ARV were showing high numbers than males in the villages. This finding corroborates two studies, one from Tanzania and the other from Malawi, which respectively found that 69% and 64% of people on ART were women (Dennis *et al*. 2007; Vilili 2008). This could partially be that many females in the villages were involved in health-related matters and often made a follow-up when they fell sick, whereas males mostly kept their illnesses to themselves. Some of the people living with HIV believed that ARV therapy could better their lives during the initial stages of introduction, but with time, they lost hope and discontinued the treatment. In a study on the progression from acquiring AIDS and until death in Botswana, it was found that 85% of the people who received ART survived in the first year and 95% survived each subsequent year (Stover, Dayton & Gabriel 2008). It was noted that first-year survival was lower than in subsequent years since some people started ART too late and died before it had a chance to restore their immune system function. This could explain the reluctance of some people on ARV in the study area because they started the treatment late.

It was also found that it was generally not the practice for parents in the study villages to communicate AIDS and ARV issues with their children. In Setswana culture, communication between parents and children about sexual behaviour is not common. This leads to children making mistakes because there is no one to guide them at home on sexual behaviour. Some of the people living with HIV were working and their workplaces did not have ARV drugs and they sometimes needed to miss work in order to refill. Some employers refused to permit their employees to go on leave; this led to some patients on ARVs defaulting on treatment. It was found that there were religions in the area which discouraged the use of ARV. The spiritual leaders of some of these religions made their followers believe that God was the only healer. There were also traditional doctors who made their patients mix traditional herbs with ARV treatment and cultural practices in infant feeding which affected ARV uptake in the study area. Generally, women who were on ARV were not advised to breastfeed if they were not on PMTCT. Breastfeeding is important in Setswana culture, and as noted, if a mother did not breastfeed her child, she was stigmatized and called all kinds of names (MOH 2008).

Even though ARV uptake had constraints at the village level, efforts were being made to overcome some of them. It was observed that nurses, doctors and chiefs taught people normally at kgotlas in the villages about the dangers of the epidemic and how to use ARV. The villagers including patients on ARV were also provided with free HIV testing and condoms in order to promote change in behaviours. The MOH provided information to the public using its strategy known as IEC. This involved giving people pamphlets such as *20 Most Asked Questions*, flyers on the importance of ARVs and interactive Community Mobilization Tool kits. In collaboration with ACHAP, the MOH had provided IDC Clinics which were attached to village hospitals and clinics. In the study area, three of the villages, Thamaga, Kumakwane and Mankgodi, were provided with such facilities.These facilities were well equipped with both human resources and ARV drugs which were dispensed freely to patients.

Encouraging progress has been made with providing facilities and remedies for people on ARV in the study villages. The main driving force behind the progress being made was the government's Masa ARV programme which provided free treatment and support given to the programme by NGOs such as ACHAP. In spite of the constraints outlined above, the outlook for ARV uptake looks generally promising for the future.
